# The Salts of Pyramidon1Deutsche Praxis vereinigt mit Zeitschrift für Prakt. Aerzte und Medizinische Neuigkeiten, 1905, No. 7.

**Published:** 1905-07

**Authors:** Franz Tauszk

**Affiliations:** University Tutor in Budapest


### TRANSLATION.

### The Salts of Pyramidon[1]

By FRANZ TAUSZK,
University Tutor in Budapest.

[Translated for the *Buffalo Medical Journal*.]

WHEN we refer to antipyresis in cases of pulmonary tuberculosis, it is only with respect to the symptomatic treatment; in no wise, however, do we believe that even by the most rational, practically managed antipyretic procedure, can we gain the slightest specific influence over the nature of pulmonary tuberculosis. I am convinced, however, that by means of a suitably administered antipyretic, according to the degree and intensity

1. Deutsche Praxis vereinigt mit Zeitschrift für Prakt. Aerzte und Medizinische Neuigkeiten, 1905, No. 7.

of the fever, we may improve and beneficially act on the vigor of the patient.

The number of drugs appearing as antipyretics in pulmonary tuberculosis is enormous. There are some good and some very good; still it is certain that among them is not one which is so good a remedy that a better might not appear. Among those preparations which, with many others, have appeared in the course of recent years and proved valuable in practice, is pyramidon.

It has been utilised particularly in cases of phthisis with elevation of temperature. At the Tuberculosis Congress in Berlin, Kobert directed the attention of physicians to pyramidon, and recommended the drug to combat the fever of tuberculosis. He based his report on his experience with nearly 6,000 cases, and came to the conclusion that with respect to action, pyramidon surpassed quinin, euchinin, antipyrin, lactophenin, antifebrin, salipyrin and tolipyrin. Kobert emphasised the fact that pyramidon influenced the heart beneficially rather than injuriously; and credited it with a stimulating effect on the appetite, and as indirectly increasing the body weight. It is not necessary to mention all those who had tried pyramidon in cases of pulmonary tuberculosis and who reported favorable results. The antipyretic as well as the antineuralgic action of the remedy has been so positively proven, that a further confirmation in this respect also appears unnecessary. We will refer, then, only briefly to the communication of Ladislaus v.Ketly, in which he states that he observed good results with pyramidon in cases where other drugs, previously tried, failed or had not reduced the temperature slowly and permanently. For the fever of tuberculosis, pyramidon may be given unhesitatingly in doses of 0.15 to 0.3 grams (2⅓ to 5 grains) without causing disagreeable symptoms.

I myself have had the opportunity in innumerable instances to study the action of pyramidon in cases of febrile and afebrile patients, and I number the same in the list of the most valuable of remedies. I also wish to state that pyramidon acts antipyretically even in small doses, and does not injuriously influence the heart. It answers, therefore, all the conditions we are justified in seeking in a good antipyretic. It is not to be denied, however, that with many patients in whom there is a tendency to profuse perspiration during the reduction of the temperature, pyramidon will produce this condition, which prevents its administration in such cases, in spite of its otherwise favorable qualities.

In order to overcome these disagreeable phenomena, the following salts of pyramidon are offered:

1. Salicylate of pyramidon (1 molecule of pyramidon and 1 molecule of salicylic acid) $C_{13}H_{17}N_3O.\ C_7H_6O_3$.

2. Acid camphorate of pyramidon (1 molecule of pyramidon and 1 molecule of camphoric acid) $C_{13}H_{17}N_3O. \quad C_{10}H_{16}O_4$.

3. Neutral camphorate of pyramidon (2 molecules of pyramidon and 1 molecule of camphoric acid $(C_{13}H_{17}N_3O_2)_2. \quad C_{10}H_{16}O_4$.

All three salts are in the form of white crystalline powder, which melt between 74°-86° C., and dissolve easily in water as well as in alcohol. Their pharmacological effect was submitted to thorough tests. Compared with that of pyramidon, the toxicity of the salicylate of pyramidon is less, while its anodyne ("pain stilling") action decidedly greater. From these pharmacological experiments it follows that the simultaneous administration of the salicylic acid and pyramidon does not produce the result that is produced by the administration of the salicylate of pyramidon.

As regards the camphorates of pyramidon, they possess simultaneously the antipyretic action of pyramidon and the antihydrotic property of the camphoric acid component. The action of these camphorates of pyramidon is modified according to the different methods of their combination, in so far that the antihydrotic effect is greater in the acid camphorate, while the antipyretic action predominates in the neutral camphorate. In both of the camphorates, the antihydrotic action of camphoric acid is in a great measure increased, and the toxicity of the pyramidon is lessened. These facts are explained in that the salicylic acid and camphoric acid salts of pyramidon are very readily soluble and easily absorbed, whereas the salicylic and camphoric acid, each alone, dissolve with great difficulty and thus are poorly adapted for absorption.

When I decided to test the therapeutic value and the practical application of the three pyramidon salts. I employed the same especially in a febrile patient suffering from neuralgia, lancinating pains of tabes dorsalis, chronic arthritis, associated with articular pains or neurasthenic cephalagia, and must declare that these salts in doses of 0.5-1.0 grams (7½ to 15 grains) once or twice daily, confirmed the hitherto acknowledged superior anodyne property of pyramidon. My observations did not fail to show that the temperature of nonfebrile patients in the first hours after taking the remedy, either remained unchanged or at the most, declined 0.3° C. In those cases where such insignificant reduction of the body heat was observed, this appeared ½ to 1 hour after the taking of the remedy, and after the course of 2 hours, the normal temperature was again reached. I did not observe any unpleasant symptoms from the stomach; in a few individual cases, I observed moderate sweating after the administration of the salicylate of pyramidon.

I further tested the pyramidon salts in different febrile diseases, being led thereto by the publication of Blumenthal of the

Stadelmann Division of the "Am Urban" Hospital, who expressed himself very favorably after detailed experiments with these salts in cases of tuberculosis. In this report, however, I desire to mention only those cases in which my observations extended to the action of the drug in cases of tuberculous fever.

Each of the three salts, the salicylate of pyramidon as well as the acid and neutral camphorates, possesses decided antipyretic action, but not, however, in equal degree. The strongest antipyretic action is furnished by the salicylate, somewhat weaker by the neutral camphorate, and the least by the acid camphorate. The size of a single dose, with which we may secure a sufficient antipyretic action, varies according to the individual; in general, however, it may be said that to obtain equal results, the average dose of the salicylate is 0.25 to 0.30 gram (4 to 5 grains), to reduce the temperature 1-2° C., of the neutral camphorate 0.50 to 0.75 gram (7½ to 12 grains) and of the acid camphorate, 0.50 to 1.0 gram (7½ to 15 grains). The reduction of the temperature begins within the first quarter of an hour after taking the remedy, progresses equally, and after two hours approximately reaches the lowest degree. From there, the temperature begins to rise gradually and reaches, after five to six hours, its original height.

My experience shows that in cases of high fever it is best to administer larger doses at once. Thus, for instance, in the case of a man 37 years old, suffering from tuberculosis, with a temperature of 38.4° C. (102° F.), complete apyrexia followed in 1½ hours after the administration of 0.25 gram (4 grains) of the salicylate of pyramidon. In the same patient, with the extension of the pulmonary process, the temperature suddenly rose to 39.2 to 39.8° C. (103 to 104° F.) after we had noted for weeks, daily afternoon temperature of 38.2 to 38.5° C. (101° to 102°F.). In this state, 0.25 gram (4 grains) of the salicylate could accomplish a reduction in temperature of only 0.4 to 0.5° C., whereas one dose of 0.50 gram (7½ grains) produced in two hours a fall in the temperature to complete apyrexia. These experiences included also the action of the camphorate of pyramidon.

One may secure very favorable results also with frequent, equally divided small doses administered daily, when it is desired to keep the patient free of fever. In cases of slight elevation in temperature, it suffices, for instance, to administer to the patient 0.25 gram (4 grains) of the salicylate three times daily at regular intervals in order to have the temperature remain at normal. The same is true of the neutral and acid camphorates of pyramidon, only that somewhat larger doses of these salts must be administered.

It must be stated, however, that one cannot depend with apodictical assurance to keep febrile patients completely free of fever by the administration of the pyramidon salts at long intervals. In many cases where the temperature did not exceed 38 to 38.5° C. 101 to 102° F.), the administration of 0.25 gram (4 grains) of the salicylate, morning, noon and night, sufficed to keep the patient free from fever. In one other case the patient was free from fever all day, whereas elevation of temperature would appear at night; nevertheless, complete apyrexia was secured in this case after the administration of a fourth dose of the salicylate. In another case, where the temperature exceeded 39° C. (102°F.), and where 0.50 gram (7½ grains) of the salicylate of pyramidon three times daily was prescribed, complete apyrexia was not secured. In such cases, the desired result was obtained only after we administered 0.30 to 0.40 gram (4½ to 6 grains) of the salicylate every two to three hours. To be sure, the maximal dose was exceeded considerably,—always a doubtful procedure in such severe cases.

Nevertheless, I can positively declare that even in these cases, in which a complete apyrexia could not be acquired because of the excessively high temperature, the fever could be lessened so much by the administration of one of the other of the pyramidon salts three to four times daily, that the patients always derived considerable benefit.

In cases in which the fever was accustomed to appear at a certain time, we always secured favorable results by the administration of the pyramidon salts one half hour before the expected appearance of the fever, when the regularly recurring fever would then either not occur, or would only be apparent by an elevation of one tenth of a degree of temperature.

It is not to be denied that the organism in certain ways accustoms itself to the pyramidon salts, and shows also individual fluctuations; generally, however, this process of the organism becoming accustomed to the pyramidon salts, develops very slowly. Should we observe, for instance, that the reduction of the temperature does not follow a certain dose as promptly as when before given, it suffices to increase the dose about 0.05 to 0.10 gram (¾ to 2 grains), to again secure for a long time the former good results. In one of my cases, one daily dose of 0.25 gram (4 grains) of the salicylate for over four weeks produced a reduction of temperature of about 2° C.; from this time, however, there appeared an ever weakening action, so that toward the end of the fifth week, the same dose only produced a temperature fall of 0.6 to 0.7° C. An increase of the dose to 0.30 gram (4½ grains again effected a deduction of 1.5° C. in the body temperature, and one dose of 0.40 gram (6 grains) even caused a fall of 2° C. in the temperature.

Untoward symptoms play a fairly secondary rôle in the clinical application of the pyramidon salts. In general, small doses are well borne by the patients, and rarely produce any disagreeable symptoms in the stomach, even though the drug be used continuously for a long time. Only in those cases where larger doses of the pyramidon salts have been administered for a very long time, have I noticed an impairment of the appetite. The gastric symptoms, however, were by no means of such importance as to preclude the further administration of the remedy.

A far more important effect, appearing after the use of the pyramidon salts and relative to which a great difference exists between the different salts, is the excretion of perspiration in the patient. The reduction of the temperature which follows the taking of the salicylate of pyramidon is sometimes accompanied by an excretion of perspiration, which is so intense that a further application, notwithstanding its otherwise favorable characteristics, must be discontinued. In several of my cases, the patient felt so exhausted as a result of the sweating, that it was only with great difficulty that I could persuade them to continue taking the salicylate of pyramidon.

As regards the camphorates of pyramidon, they differ in this respect not alone essentially and favorably from the salicylate, but also from all other antipyretics which are employed in tuberculosis. Indeed, they offer decided advantages over these remedies, in that their administration is accompanied by scarcely apparent or only very insignificant excretion of sweat. Between the two camphorates of pyramidon there exists graduated differences, in so far that the acid camphorate exercises, in addition to the antipyretic, a decidedly stronger antihydrotic action than the neutral camphorate. A new indication as regards the application of the pyramidon salts is thereby apparent. My observations in this regard demonstrate that the acid camphorate should be regarded as a very reliable antipyretic and antihydrotic in those cases where the patients show an inclination to excessive sweating. It is to be stated too, that the pyramidon salts beneficially influence the condition known as "air hunger," a characteristic absent in other antipyretics.

In none of my cases, could I discover an injurious effect of the pyramidon salts on the circulation, especially on the heart, even not in those febrile patients whose condition was quite bad and whose heart function was weak.

In addition, the pyramidon salts possess very beneficial characteristics which, in appropriate cases, we designate as side effects

—that is, its decided "pain stilling" action. I had an opportunity to observe this beneficial effect on a patient with pulmonary tuberculosis by whom acute rheumatic polyarthritis developed, as well as on a patient ill with lumbago. The pain stilling quality of the salicylate proved quite satisfactory in both cases.

As a special indication for the application of the salicylate of pyramidon, I desire to cite those cases where pulmonary tuberculosis is complicated with pleuritis or with pleuritic exudation. In these cases I had the opportunity to observe not only the antipyretic but also the diuretic action of the salicylate. One of these cases was a woman with a beginning pulmonary tuberculosis, in whom there developed a pleuritic exudate which extended up to the third rib on the right side. The patient was given 0.50 gram (7½ grains) of the salicylate of pyramidon three times daily. The daily amount of the urine excreted gradually increased from 600 to 800 c.cm. to 2 to 2½ litres, through which, by continued treatment, the exudate disappeared in a little more than two weeks. Besides the diuresis, the excessive sweating effected the diminution of the exudation. A second case concerned that of a young man, 24 years old, with pulmonary tuberculosis in an advanced stage, in whom the administration of 0.5 gram (7½ grains) of the salicylate of pyramidon three times daily, completely resolved the exudate in 8 to 10 days. I regard it advisable to try large doses of the salicylate of pyramidon in all varieties of pleuritic exudation. This remedy should also be administered especially in those cases in which a febrile process of the lungs is associated with painful complications.

I sum up my observations as follows:

1. The pyramidon salts are valuable antipyretics, and as regards this antipyretic action, the salicylate surpasses both the camphorates of pyramidon.

2. The salicylate displays its antipyretic action in one dose of 0.25 to 0.50 gram (4 to 7½ grains), and the camphorates in 0.50 to 1.0 gram (7½ to 15 grains) pro dosi administered three times daily.

3. The salicylate of pyramidon may be employed in cases of pleuritic exudation.

4. All three pyramidon salts possess equal "pain stilling" qualities, and produce scarcely any untoward symptoms.

5. The acid camphorate exercises its temperature reducing effect without being followed by excessive sweating, and because of its antihydrotic action should be considered one of the most important of the prominent antipyretic remedies. Consequently it may be employed with advantage in cases of pulmonary tuberculosis accompanied by excessive secretion of sweat.

